# Is vaginal progesterone treatment associated with the development of gestational diabetes? A retrospective case–control study

**DOI:** 10.1007/s00404-018-4895-1

**Published:** 2018-09-17

**Authors:** Klara Rosta, Johannes Ott, Fanni Kelemen, Wilhelm Temsch, Tobias Lahner, Theresa Reischer, Hanns Helmer, Aniko Somogyi

**Affiliations:** 1 Department of Obstetrics and Gynecology, Medical University of Vienna, Waehringer Guertel 18-20, 1090 Vienna, Austria; 2 University of Szeged, 12 Dóm tér, 6720 Szeged, Hungary; 3 Center for Medical Statistic and Informatics and Intelligent Systems, Medical University of Vienna, Waehringer Guertel 18-20, 1090 Vienna, Austria; 4 2nd Department of Internal Medicine, Semmelweis University, Szentkirályi u.46, Budapest, Hungary

**Keywords:** Pregnancy, Gestational diabetes mellitus, Preterm birth, Prenatal care, High-risk pregnancy

## Abstract

**Purpose:**

To determine the incidence of gestational diabetes mellitus (GDM) in pregnant women who received vaginal progesterone due to short cervical length or to prevent recurrent preterm birth.

**Methods:**

In this retrospective study, we included 190 women with singleton pregnancies at risk for preterm birth who received vaginal natural progesterone (200 mg daily between gestational weeks 16 + 0 and 36 + 0) for a minimum of 4 weeks and delivered > 28 weeks. The control group consisted of 242 age- and body mass index (BMI)-matched patients without progesterone administration. Data were acquired from a database containing prospectively collected information. Patients with pre-existing diabetes, and conception after in vitro fertilisation procedure were excluded.

**Results:**

The incidence of GDM did not differ significantly between the progesterone-treated and the control group (14.7% vs. 16.9%, respectively; *p* = 0.597). In a binary regression model, patients with higher pre-pregnancy BMI (OR 1.1; *p* = 0.006), and those with a family history of diabetes had a higher risk for GDM development (OR 1.8; *p* = 0.040), whereas vaginal progesterone treatment had no significant influence (*p* = 0.580).

**Conclusion:**

The use of vaginal progesterone for the prevention of recurrent preterm delivery and in women with a short cervix does not seem to be associated with an increased risk of GDM.

## Introduction

Gestational diabetes mellitus (GDM), defined as glucose intolerance, first recognized during pregnancy is one of the most common complications during pregnancy. GDM is of high clinical relevance with possible severe adverse outcomes for both mother and child during pregnancy, at childbirth and later in life, which includes increased risks of preeclampsia, perinatal morbidity, type 2 diabetes and cardiovascular disease [1]. Estimates of GDM indicate that it is a common disease with a reported prevalence of 2–6% in Europe, although rates may be even higher (e.g. > 10% in Austria), depending on the selected population and diagnostic criteria applied [2]. Moreover, increasing trends in the prevalence of GDM have been reported in several Western countries, mainly attributed to delayed maternal age at pregnancy as well as to the obesity epidemic. This seems reasonable since advanced maternal age at pregnancy, pre-pregnancy overweight or obesity belongs to the most important risk factors for GDM [3].

Progestogens (including synthetic progesterone analogues and natural progesterones) are used worldwide to prevent preterm birth, a leading cause of perinatal mortality and morbidity. It has been demonstrated that progesterone is efficient in terms of prolonging pregnancy and improving neonatal morbidity and mortality [4–6]. The use of either synthetic or natural progestogens as a measure to prevent preterm birth has increased substantially in the last several years [7]. However, it has been suggested that progesterone might exert diabetogenic effects [8].

Although the diabetogenic effects of progesterone supplementation have been investigated in in vitro [9, 10] as well as in retrospective [8, 11, 12] and prospective [13–15] studies, the results remain inconclusive. Women with an increased risk for preterm delivery due to shortened cervical length with a transvaginal ultrasound scan carried out between 16 + 0 and 24 + 0 weeks of pregnancy showing a cervical length of < 25 mm and/or history of a previous preterm birth in our department routinely receive vaginal progesterone treatment starting at 16–20 weeks of gestation until 36 weeks of gestation in an attempt to prolong pregnancy. This allowed us to study the effect of vaginally applied natural progesterone on the incidence of gestational diabetes mellitus which, to the best of our knowledge, has not been studied before. Accordingly, in this retrospective case–control study, we aimed to compare the incidence of GDM between women who had been treated with vaginal progesterone with a matched control group consisting of untreated women.

## Materials and methods

### Patient population and study design

For this retrospective study, we selected women from a databank created within the framework of a screening program for pregnant women at perceived risk for preterm delivery at the Department of Obstetrics and Gynecology of the Medical University of Vienna, a tertiary centre for fetomaternal medicine in eastern Austria [16, 17]. Patients at risk of preterm birth had either a history of one or more spontaneous preterm deliveries or mid-trimester pregnancy losses between 16 + 0 and 34 + 0 weeks of pregnancy and/or shortened cervical length of < 25 mm in a transvaginal ultrasound scan performed between 16 + 0 and 24 + 0 weeks of pregnancy. 387 women were identified between January 2011 to July 2017, who received vaginal progesterone (200 mg/ day) for a minimum of 4 weeks from the second trimester of their pregnancy. Three hundred age- and BMI-matched pregnant women without progesterone treatment were selected as the control group. For the case and the control groups the following exclusion criteria were applied: pre-existent diabetes mellitus; multiple pregnancies; women with ongoing pregnancies; women with deliveries < 28 + 0 weeks; women with a pre-pregnancy BMI > 37, since the extremely high pre-pregnancy BMI poses a known risk for GDM [18] and often appear together with polycystic ovary syndrome [19, 20]. Moreover, since no data about early oral glucose tolerance tests (OGTTs) were available, we aimed to exclude the majority of early GDM cases. According to the follow-up analysis of the DALI study, the average BMI of women who developed early GDM was 36.2 kg/m^2^ [18]. Only three women in the vaginal progesterone treatment group were excluded by applying this criterion, women who had conceived using artificial reproduction treatments, since the latter have been reported to increase the risk of GDM [21–23]. Since progesterone is often provided as high-dose luteal support to women after artificial reproduction treatment, women who were treated for less than 4 weeks with vaginal progesterone, and patients with incomplete data were also excluded from the study. After application of these exclusion criteria, 190 women were finally included in the case group and 242 women in the control group.

### Data quality and management

We used the ViewPoint Fetal Database 5.6.9.17.^®^ software which contains prospectively collected information for data retrieval. For validation of accuracy, we looked at the distribution and ranges of our data. Extreme values were double-checked using source files. In addition, we conducted random checks by two independent investigators to ensure the accuracy of our data. The main outcome measure was the incidence of the diagnosis of GDM. In Austria, GDM is defined as an abnormal 75 mg OGTT performed from weeks 24 + 0 to 28 + 0 according to the Austrian national guideline and Hyperglycemia and Adverse Pregnancy Outcomes (HAPO) Criteria [24] or elevated blood sugar (> 140 mg/dl postprandial or > 95 mg/dl fasting values) at measurements performed later in pregnancy [19]. In addition, the following data were collected: maternal age, pre-pregnancy body mass index (BMI), family history of diabetes mellitus, gravidity and parity, information on whether the women had suffered from GDM in a previous pregnancy, use of betamethasone for lung maturation and tocolysis with atosiban in the evaluated pregnancy, since these medications are considered to influence GDM development [25], gestational age and mode of delivery and birthweight. The study was approved by the Institutional Review Board of the Medical University of Vienna (IRB number: 2191/2017).

### Routine care

Patients with shortened cervical length < 25 mm in a transvaginal ultrasound scan between 16 + 0 and 24 + 0 weeks of pregnancy and/or a history of preterm birth underwent regular follow-up examinations for cervical length measurement at our department every 2 weeks until week 34 + 0. Patients in the control group had a follow-up visit at gestational week 34 for exact biometry and evaluating the delivery mode and at the onset of labour or at week 40 + 0 at the latest. Both the case and the control group underwent regular examinations at a private gynaecologist and obstetrician, in weeks 18–22, and 30–34. These follow-up examinations included foetal biometry, evaluation of the placenta, amniotic fluid, dip stick urine test, including glucose, blood tests for virology, blood group, and OGTTs between gestational weeks (gw) 24 and –28 with 75 g oral glucose load. Concerning late-onset GDM, cases were detected based on clinical signs including glycosuria, polyhydramnion, disproportional growth or foetal abdominal circumference, which are according to guidelines used in German-speaking countries [19]. Patients with these abnormalities were instructed in blood glucose self-monitoring with a four-point blood sugar profile. If the blood sugar profile exceeded the normal values, the patient was diagnosed with GDM. In Austria, pregnant women are required to attend regular appointments in the outpatient care system. Thus, it is likely that any abnormalities between weeks 30 and 34 were recognized and the affected patients referred to their delivery center. Thereby, cases of late GDM development, i.e. after the routine OGTTs, should have become equally evident in the case and the control groups.

### Statistical analysis

Nominal variables are reported as numbers and frequencies, and continuous variables as mean values and standard deviations (SD). Statistical analysis was performed using Pearson’s Chi-squared test, Fisher’s exact test, and Student’s *t* test, as appropriate on the basis of data distributions to compare differences between the control and the progesterone-treated groups. As pre-pregnancy BMI, family history of diabetes mellitus, tocolysis, and lung maturation with betamethasone are considered to influence the development of gestational diabetes mellitus, multivariate logistic regression was tested to assess relative independent associations on the dependent outcome of GDM incidence. For this analysis, odds ratios (OR), 95% confidence intervals (95% CI) and* p*-values of the likelihood ratio test were calculated. Statistical analysis including matching was performed using the open-source statistical package, IBM SPSS 23. Differences were considered statistically significant if *p* < 0.05.

## Results

Detailed characteristics about the included pregnancies are provided in Table 1. The mean treatment duration with vaginal progesterone was 135 days (range 91–140). Betamethasone for lung maturation as well as tocolysis with atosiban was administered more often in patients with progesterone therapy (48.9% vs. 23.6%, and 48.9% vs. 17.7%, respectively, both *p* ≤ 0.001). Gestational age at delivery and the rate of preterm delivery before the 37th gestational week differed significantly between the progesterone-treated and control groups (37.43 ± 3.88 vs. 38.66 ± 3.54 weeks; *p* ≤ 0.001 and delivery before the 37th week of gestation 34.7% vs. 25.6%; *p* = 0.039). We found no other difference in patient and pregnancy parameters between the two groups.

**Table 1:** Basic patient and pregnancy characteristics of the case and control groups

	Women treated with vaginal progesterone (*n* = 190)	Control group (*n* = 242)	*p* value
Maternal age (years)*	31.46 ± 5.91	31.22 ± 5.51	0.658
Maternal age > 37 years^#^	30 (15.8%)	35 (14.5%)	0.702
Pre-pregnancy BMI (kg/m^2^)*	24.0 ± -4.72	24.16 + -4.28	0.718
Previous GDM^#^	24 (12.6%)	43 (17.7%)	0.143
Use of atosiban for tocolysis^#^	93(48.9%)	43 (17.7%)	< 0.001
Use of betamethasone for lung maturation^#^	93 (48.9%)	57(23.6%)	< 0.001
Gestational age at delivery (completed weeks)*	37.43 ± 3.88	38.66 ± 3.54	< 0.001
Preterm delivery < 37 + 0^#^	66 (34.7%)	62 (25.6%)	0.039
Preterm delivery < 35 + 0^#^	45 (23.7%)	47 (19.4%)	0.283
Preterm delivery < 32 + 0^#^	24 (12.6%)	23 (9.5%)	0.300
Delivery by caesarean section^#^	79(43.9%)	108(44.6%)	0.525
Birthweight (g)*	3004 ± 767	2996 ± 869	0.917

Data are presented as *mean ± standard deviation or ^# ^numbers (percentage)

*BMI* body mass index, *GDM* gestational diabetes mellitus

The incidence of GDM did not significantly differ between women with vaginal progesterone treatment and the control group (progesterone-treated vs. control 28/190, 14.7% vs. 41/242 16.9%, respectively; *p* = 0.597). However, due to the differences in patient and pregnancy characteristics between the case and the control groups, especially concerning the rates of lung maturation and tocolysis, a logistic regression model was used to assess the relative independent predictors for the development of GDM (Table 2). Patients with higher pre-pregnancy BMI (OR 1.1; *p* = 0.006), and those with a family history of diabetes had a higher risk for GDM development (OR 1.8; *p* = 0.040). Vaginal progesterone treatment had no significant influence (*p* = 0.580).

**Table 2:** Results of the binary logistic regression model for the prediction of GDM development

	OR (95% CI)	*p* value
Vaginal progesterone treatment	0.85 (95% CI 0.47–1.52)	0.580
Pre-pregnancy BMI kg/m^2^	1.1 (95% CI 1.02–1.15)	0.006
Family history of diabetes	1.8 (95% CI 1.03–3.14)	0.040
Betamethasone 12 mg 2 × within 24 h	1.47 (95% CI 0.51–4.22)	0.471
Atosiban* for at least 48 h	0.79 (95% CI 0.26–2.41)	0.683

*BMI* body mass index, *OR* odds ratio, *95% CI* 95% confidence interval

*In the atosiban group, women received a bolus injection of 6.75 mg intravenous in 1 min, followed by 18 mg/h for 3 h, followed by a maintenance dosage of 6 mg/h for 45 h

In a next step, the study population was divided into BMI subgroups. Again, no differences in the incidence of GDM were found between progesterone-treated and control women: neither in those with a BMI 20.0–24.9 kg/m^2^ (6/77, 7.8% vs. 15/103, 14.5%, respectively; *p* = 0.162), nor in those with a BMI 25.0–29.9 kg/m^2^ (9/40, 22.5% vs. 13/57, 22.8%, respectively; *p* = 0.972), nor in those with a BMI ≥ 30 kg/m^2^ (7/26, 26.9% vs. 20/42, 47.6%, respectively; *p* = 0.090).

## Discussion

In this retrospective case–control study, the use of vaginal progesterone was not associated with an increased risk of GDM development. This is of clinical importance, since the use of vaginal progesterone for the prevention of preterm birth and for reducing the risk of preterm delivery is widespread and recommended by international guidelines. Notably, the American College of Obstetricians and Gynecologists and SMFM recommended treatment with either 17 OHP-C or vaginal progesterone for women with a prior spontaneous preterm birth to prevent recurrent preterm birth [26]. According to the National Institute for Health and Care Excellence Guidelines, either cervical cerclage or vaginally administered progesterone can be administered in women with a history of previous preterm birth or a shortened cervix [27]. The 2017 SMFM statement reaffirmed that vaginal progesterone should not be considered a substitute for 17 OHP-C in women with a singleton gestation and a history of prior spontaneous preterm birth and proposed the use of vaginal progesterone suppositories (200 mg) or progesterone gel (90 mg) in singleton pregnancies with no known preterm birth history and shortened cervix (< 20 mm) [7].

However, the high incidence of gestational diabetes in our patient collective (progesterone-treated vs. control 28/190, 14.7% vs. 41/242 16.9%, respectively; *p* ≤ 0.597) mirrors the progressive patient care at the Medical University of Vienna and the accumulation of GDM patients as high-risk pregnancies at our institution. In Austria the HAPO criteria are used for screening of GDM [19]. The incidence of GDM in our study is comparable to the overall frequency of GDM (17.8%, range 9.3–25.5%) detected among the 15 centres that participated in the HAPO Study using the new International Association of the Diabetes and Pregnancy Study Groups (IADPSG) criteria [28]. The prevalence of gestational diabetes varies between 1% and 39% depending on the specific population, ethnicity and diagnostic criteria used [18, 28–30]. As preterm birth poses a burden on families, primary prevention is quintessential.

Progesterone compounds can be administered orally, intramuscularly and vaginally. Studies on the diabetogenic effect of progesterone supplementation have yielded inconclusive results. A number of studies have suggested that intramuscular administration of 17-alpha hydroxyprogesterone caproate (17 OHP-C) might pose a risk of increasing the incidence of GDM [8, 11–13]. On the other hand, a secondary analysis of two randomized placebo-controlled trials of 17 OHP-C found no association with higher rates of GDM in either group [14]. Intramuscularly administered progesterone might affect carbohydrate metabolism in the second trimester of the pregnancy, when progesterone levels are high. Vaginal progesterone might influence carbohydrate metabolism to a lesser extent, since it mainly exerts a local effect on the cervical tissue. Although serum levels of progesterone were not recorded in our study, increase in serum levels after topical application has not been reported [31]. To our knowledge this is the first study to evaluate the possible diabetogenic effect of vaginal progesterone administration on the incidence of GDM.

It could be postulated that the diabetogenic effect of progesterone might be critical in subpopulations with risk factors for GDM. Although our study population was matched for BMI, we divided the study population into BMI subgroups to compare the diabetogenic effect of vaginal progesterone within these subgroups. In contrast to the study of Rebarber et al. [13] we found no difference in the incidence of GDM when comparing the BMI subgroups of progesterone-treated patients and controls. BMI was also included in the binary logistic regression model for the prediction of GDM development and, as expected, patients with higher pre-pregnancy BMI had a higher risk of developing GDM.

Our study is limited by a relatively low sample size. A further limitation of the study is that the case group underwent regular control check-ups every 2 weeks, thus, there might be an observational bias to diagnose GDM after regular OGTT screening based on signs such as glycosuria, polyhydramnion, disproportional growth or foetal abdominal circumference [19]. Nonetheless, the Austrian outpatient care system requires a detailed ultrasound examination between gestational weeks 30 and 34 which functions as a checkpoint to recognize any signs of pathology and refer the patient to the delivery center. Our results reflect a good functioning outpatient care system, since the incidence of GDM in the control group did not seem to be underdiagnosed, compared to international standards. Furthermore, this study did not aim to assess the efficacy of vaginal progesterone treatment in the prevention of preterm birth, and we did not aim to fully evaluate foetal outcomes. We were also unable to stratify the data by maternal race or ethnicity, since these data were not routinely recorded in the database.

In conclusion, our data suggest that vaginal progesterone treatment for cervical shortening/risk of preterm birth does not lead to an increased rate of GDM development, at least in women with a BMI < 37.0 kg/m^2^ who had conceived naturally. However, further studies are needed to evaluate the diabetogenic effect of widely used natural vaginal progesterone on high-risk populations for GDM.
